# Lumen–Stent Mismatch Affects Long-Term Strut Healing After Primary PCI of Left Main Lesions: An Exploratory Follow-Up OCT Study

**DOI:** 10.3390/diagnostics16101519

**Published:** 2026-05-17

**Authors:** Zlatko Mehmedbegovic, Vladan Vukcevic, Sinisa Stojkovic, Branko Beleslin, Dejan Orlic, Miodrag Dikic, Dejan Milasinovic, Milorad Tesic, Srdjan Aleksandric, Vladimir Dedovic, Milorad Zivkovic, Stefan Juricic, Dario Jelic, Djordje Mladenovic, Lazar Travica, Damjan Simic, Djordje Dukic, David Sarenac, Marko Ristic, Dijana Bojovic, Biljana Milicic, Goran Stankovic

**Affiliations:** 1 Department of Cardiology, University Clinical Center of Serbia, 11000 Belgrade, Serbia; zlatkombegovic@gmail.com (Z.M.); vladan.vukcevic@gmail.com (V.V.); sstojkovi@open.telekom.rs (S.S.); branko.beleslin@gmail.com (B.B.); orlicmail@yahoo.com (D.O.); miodrag.dikic@gmail.com (M.D.); milasin.d18@gmail.com (D.M.); misa.tesic@gmail.com (M.T.); srdjanaleksandric@gmail.com (S.A.); vladeda@gmail.com (V.D.); mzivkovic05@hotmail.com (M.Z.); stefan.juricic@gmail.com (S.J.); duga13@gmail.com (D.J.); mladendjolem@gmail.com (D.M.); lazartravica18@gmail.com (L.T.); simicdamjan@hotmail.com (D.S.); djoleczv1994@gmail.com (D.D.); davidsarenac1604@gmail.com (D.S.); marko.ris@live.com (M.R.); dijanabojovic8@gmail.com (D.B.); 2 Faculty of Medicine, University of Belgrade, 11000 Belgrade, Serbia; 3 School of Dental Medicine, University of Belgrade, 11000 Belgrade, Serbia

**Keywords:** optical coherence tomography, unprotected left main, percutaneous coronary intervention, stent malapposition, strut coverage, lumen–stent mismatch

## Abstract

**Background**: Long-term stent healing after primary PCI of culprit unprotected left main (ULM) lesions is insufficiently explored. In this setting, large vessel size and bifurcation anatomy may limit angiographic stent optimization and contribute to persistent strut malapposition and incomplete coverage. **Objectives**: To identify OCT-derived geometric and healing parameters associated with long-term strut coverage and malapposition after angiography-guided primary PCI of culprit ULM lesions. **Methods**: This single-center exploratory study included 30 patients with long-term OCT follow-up after angiography-guided primary PCI of culprit ULM lesions. OCT analysis was performed separately in three prespecified subsegments: the left main (LM), polygon of confluence (POC), and distal main branch (dMB). Five predefined strut-level healing outcomes were analysed: covered struts, malapposed struts, malapposed and uncovered struts, significantly malapposed struts (>400 μm), and significantly malapposed and uncovered struts. Associations between patient-level healing outcomes and OCT-derived measures of lumen geometry, stent dimensions, neointimal response, and an exploratory lumen–stent mismatch variable were assessed using univariable and multivariable linear regression. **Results**: A total of 31,703 struts were analysed. Overall strut coverage was 90.7 ± 6.6%. Compared with the dMB, proximal ULM segments (LM and POC) showed lower strut coverage (82.8% and 84.2% vs. 93.9%, *p* < 0.001) and higher malapposition rates (17.4% and 14.2% vs. 0.4%, *p* < 0.001). In regression analysis, larger native lumen dimensions were associated with lower strut coverage and higher malapposition, whereas larger achieved stent area was associated with better strut healing. The exploratory lumen–stent mismatch variable in multivariable models with all five healing outcomes in multivariable models (all *p* < 0.01). **Conclusions**: After angiography-guided primary PCI of culprit unprotected left main lesions, long-term strut healing was significantly influenced by the mismatch between native reference lumen area and the achieved mean stent area. Whether intravascular imaging–guided optimization of stent sizing and expansion in large-calibre left main anatomy improves strut healing requires further investigation.

## 1. Introduction

Percutaneous coronary intervention (PCI) of culprit unprotected left main (ULM) lesions in acute coronary syndrome (ACS) is a high-risk procedure performed in a large-caliber bifurcation vessel. In this setting, angiographic procedural success does not necessarily reflect optimal stent expansion, apposition, or subsequent vascular healing [1,2]. Optical coherence tomography (OCT) enables direct assessment of strut coverage, malapposition, and neointimal response. In non-left main lesions, serial OCT studies have shown that strut coverage is usually near-complete within the first months after drug-eluting stent implantation. By contrast, long-term OCT data after PCI of culprit ULM lesions remain limited [3]. Available pathological and OCT studies suggest that uncovered and malapposed struts occur more often in left main (LM) bifurcation segments than in distal coronary segments [4,5].

However, the geometric determinants of long-term strut healing in culprit ULM lesions remain poorly defined. It is unclear whether the relation between native lumen size and achieved stent dimensions is associated with subsequent healing patterns. In our prior pilot OCT study, proximal left main segments showed less complete healing at follow-up, but the sample size was insufficient for a more detailed mechanistic analysis [6]. The present study was therefore designed as an exploratory OCT analysis to assess long-term strut coverage and malapposition after angiography-guided primary PCI of culprit ULM lesions, with particular focus on geometric correlates related to native lumen size and achieved stent dimensions.

## 2. Materials and Methods

### 2.1. Study Population and Design

**Figure 1:**
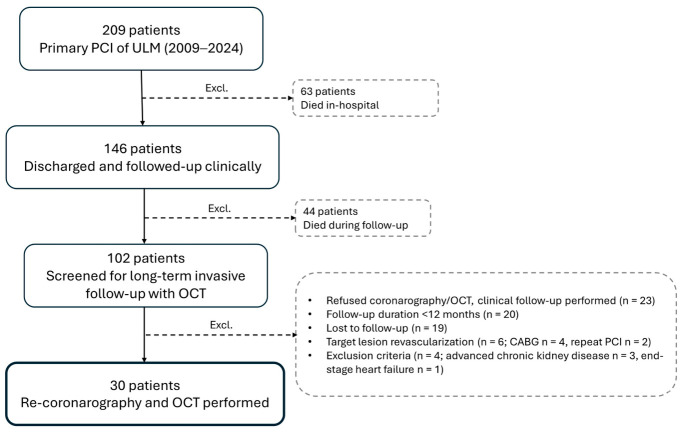
Patient flow-chart. CABG—coronary artery bypass grafting; OCT—optical coherence tomography; PCI—percutaneous coronary intervention; ULM—unprotected left main coronary artery.

This was a single-center exploratory observational study of 30 consecutive hospital survivors treated with angiography-guided primary PCI for culprit ULM lesions between 2009 and 2024, with long-term OCT follow-up performed at 4.4 ± 3.6 years. The study design required a minimum follow-up duration of 12 months, corresponding to the expected completion of the biological healing response after drug-eluting stent implantation. As presented in Figure 1, patients with follow-up < 12 months, target lesion reintervention during follow-up, refusal of invasive follow-up, loss to follow-up, or end-stage organ disease were excluded (82 patients in total).

The study protocol was approved by the Council of the Scientific Field of Medical Sciences, Faculty of Medicine, University of Belgrade (No. 61206-4744/2-21), and by the Research Board of the Department of Cardiology, University Clinical Center of Serbia (review No. 1883). All participants provided written informed consent. The study was conducted in accordance with the Declaration of Helsinki.

### 2.2. PCI Procedure and OCT Acquisition

Index PCI was performed according to contemporary clinical practice in the acute setting. Follow-up OCT imaging was performed using a frequency-domain coronary OCT system according to consensus standards for acquisition, measurement, and reporting of intravascular OCT studies [7]. Intracoronary nitrates were administered before imaging to reduce vasomotor tone and improve lumen delineation. Automated pullback was performed after contrast-mediated blood clearance, with Z-offset calibration before acquisition. Pullbacks were analysed at 0.2 mm intervals. Before quantitative analysis, all pullbacks were systematically reviewed for image quality. Pullbacks with incomplete blood clearance, severe motion artifact, catheter decentring, or inadequate visualization of stent struts were excluded from quantitative analysis. This approach was prespecified to ensure reliable strut-level measurements, particularly in large-calibre proximal left main segments, where image quality may affect border delineation and apposition assessment.

### 2.3. OCT Analysis and Measurements

OCT image analysis was performed offline using dedicated software (CAAS Workstation 7.3, Pie Medical Imaging, Maastricht, The Netherlands) by experienced analysts (ZM, DJ) blinded to clinical data. Analysis followed established OCT standards for coronary stent assessment [7]. Struts were classified as covered only when neointimal tissue completely covered the luminal strut surface. Partially covered struts were classified as uncovered. Malapposition was defined as a strut-to-wall distance exceeding the nominal strut plus polymer thickness, in accordance with consensus OCT methodology for coronary stent analysis.

For bifurcation analysis, the treated segment was divided into three prespecified subsegments: left main (LM) as proximal main branch, polygon of confluence (POC) as bifurcation segment, and proximal LAD as distal main branch (dMB). This segmental approach was used because bifurcation geometry differs across these regions and may affect strut apposition and coverage. Segment definition and reporting were based on accepted bifurcation concepts and prior consensus documents on bifurcation imaging and analysis [5,8].

The following OCT-derived geometric and healing variables were measured at the patient level: reference lumen diameter, reference lumen area, minimal lumen area (MLA), mean lumen area, percent lumen stenosis, minimal stent area (MSA), mean stent area, minimum stent expansion, mean stent expansion, lumen volume, stent volume, neointimal hyperplasia (NIH) volume, and malapposition volume. These variables were selected because they are routinely obtainable from coronary OCT pullbacks and directly describe native lumen geometry, achieved stent dimensions, and tissue healing.

**Figure 2:**
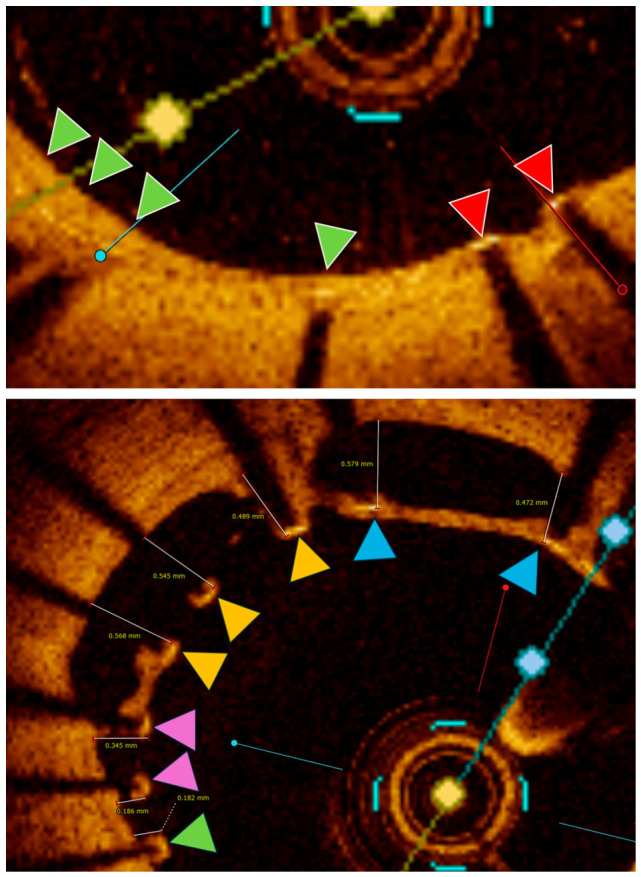
OCT strut analysis. Upper panel: Strut coverage analysis in embedded struts; Green triangle—covered; red triangle—uncovered (partially covered struts); Lower panel: Strut coverage analysis in malapposed struts. Green triangle—malapposed and covered; pink triangle—malapposed and uncovered; yellow triangle—significantly malapposed (>400 µm) and uncovered; blue triangles—significantly malapposed (>400 µm) and covered.

Strut-level analysis was performed at each cross-section. Individual struts were classified according to coverage status (covered or uncovered) and apposition status relative to the vessel wall. Malapposition was defined as a measured strut-to-wall distance exceeding strut thickness plus polymer thickness. Significant malapposition was defined as a strut-to-wall distance >400 μm (Figure 2). This threshold was selected based on prior OCT and pathological studies demonstrating that larger separation distances are less likely to resolve over time and are more frequently associated with persistent uncovered struts [9,10]. The adoption of a >400 μm threshold facilitated a distinction between minor malapposition, which, according to prior longitudinal evidence, is frequently amenable to ‘late correction’ via adaptive vessel remodeling or neointimal proliferation, and more severe distances that have established biological significance and are linked with persistent lack of strut coverage [11,12,13,14].

Inter-observer and intra-observer reproducibility were assessed in a random 10% subset of the dataset. Agreement for strut coverage and apposition classification was quantified using Cohen’s kappa (See Appendix A.1).

### 2.4. Exploratory OCT-Derived Parameter of Lumen–Stent Mismatch

Because native lumen and stent dimensions were correlated, we defined an exploratory composite variable (reference lumen area − mean stent area) to reflect the relationship between vessel size and achieved stent expansion. In the absence of procedural OCT imaging, lumen–stent mismatch was derived from follow-up OCT as a geometric descriptor of the relationship between native vessel size and achieved stent area, rather than actual procedural stent sizing and lumen measurements at the time of the index PCI. This variable was additionally used to reduce collinearity when lumen- and stent-related variables were examined in separate multivariable models.

### 2.5. Study Endpoints

Five predefined strut-level healing outcomes were analysed as dependent variables:

1. percentage of covered struts;
2. percentage of malapposed struts;
3. percentage of malapposed and uncovered struts;
4. percentage of significantly malapposed struts (>400 μm);
5. percentage of significantly malapposed and uncovered struts.

### 2.6. Statistical Analysis

Continuous variables are presented as mean ± standard deviation or median with interquartile range, as appropriate, and categorical variables as counts and percentages. Segmental comparisons were performed using repeated-measures analyses to account for within-patient clustering of subsegment measurements. Associations between OCT-derived parameters and patient-level strut healing outcomes were first assessed using univariable linear regression. Multivariable linear regression models were then constructed for each healing endpoint. Candidate predictors were selected from significant univariable associations (*p* < 0.25) and assessed for biological plausibility and collinearity before model construction. All selected variables were entered simultaneously into the multivariable models using the forced entry method. Given the limited sample size, the number of predictors included in each multivariable model was restricted where possible. Several OCT-derived variables were retained despite potential collinearity (confirmed by VIF diagnostics), as they represent complementary aspects of vessel geometry and vascular healing. This approach was chosen to preserve biological interpretability in an exploratory setting, prioritizing clinical relevance over strict statistical independence. In addition, an exploratory composite variable (lumen–stent mismatch) was introduced to capture the relationship between vessel size and achieved stent expansion while reducing redundancy between closely related variables. Model performance was summarized using the coefficient of determination (*R*^2^). Given this interdependence, multivariable models were interpreted as descriptive of interrelated mechanisms rather than as identifying independent causal predictors. This framework was essential to ensure the findings remained clinically grounded within the exploratory and hypothesis-generating nature of the study. Accordingly, no adjustment for multiple comparisons was applied, and results should be interpreted within stated limitations.

## 3. Results

### 3.1. Study Population

Thirty patients with long-term OCT follow-up after angiography-guided primary PCI of culprit ULM lesions were included. Mean follow-up duration was 1623 ± 1326 days. Most procedures were performed using a one-stent strategy, with a final angiographic success in 29 of 30 (97%) (Table 1). Additional clinical, angiographic, and procedural details are provided in the Appendix A.1 (Table A1, Table A2, Table A3, Table A4, Table A5 and Table A6).

### 3.2. OCT Dataset and Strut-Level Analysis

The mean analysed subsegment length was 6.7 ± 4.4 mm for the LM, 2.4 ± 0.9 mm for the POC, and 13.8 ± 6.7 mm for the dMB, while the mean total analysed stented segment length was 21.9 ± 6.4 mm. A total of 31,703 stent struts were analysed, corresponding to 1056.8 ± 570.3 struts per patient (Table 2).

**Figure 3:**
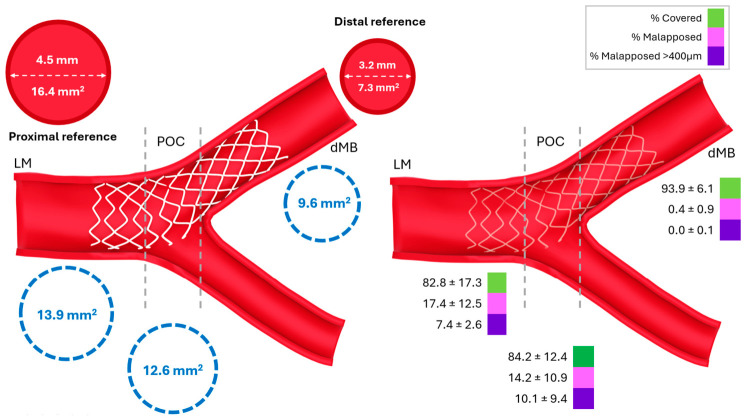
Segment-specific vessel–stent geometry (**left**) and strut healing (**right**) after angiography-guided primary PCI of culprit unprotected left main lesions.

Overall strut coverage was 90.7 ± 6.6%. Compared with the dMB, proximal ULM segments (LM and POC) showed lower strut coverage (82.8% and 84.2% vs. 93.9%; *p* < 0.001) and higher malapposition rates (17.4% and 14.2% vs. 0.4%; *p* < 0.001) (all *p* < 0.001; Figure 3 and Table 2).

The schematic illustrates the three prespecified subsegments used for OCT analysis: left main (LM), polygon of confluence (POC), and distal main branch (dMB). Red circles indicate reference vessel dimensions, and dashed blue circles indicate mean stent area. The right panel shows segment-specific percentages of covered struts, all malapposed struts, and significantly malapposed struts (>400 μm) at follow-up. Proximal segments showed lower strut coverage and more frequent malapposition-related abnormalities than the dMB. (The figure is schematic and derived from OCT analysis, not a direct OCT image.)

LM, left main; POC, polygon of confluence; dMB, distal main branch; μm, micrometers.

Significantly malapposed struts (>400 μm) were also more frequent in proximal segments (7.4% and 10.1% vs. 0.0%; *p* < 0.001). Among all malapposed struts, uncovered status was more common at malapposition distances >400 μm than at distances ≤400 μm (73.4 ± 29.2% vs. 57.7 ± 39.6%; *p* = 0.036). Detailed qualitative OCT findings and complete strut-level characterization are provided in the Appendix A.1 (Table A7 and Table A8).

Lumen and stent geometry also differed significantly across three segments (Table 3). Proximal segments had larger lumen dimensions than distal segments. Although absolute stent dimensions were also larger proximally, relative stent expansion was lower and malapposition volume was greater in proximal segments than in the distal main branch (*p* < 0.001 for overall segmental comparisons). The complete geometric and volumetric OCT dataset is provided in the Appendix A.1 (Table A9).

### 3.3. Regression Analyses of OCT-Derived Parameters for Prediction of Strut-Level Healing Outcomes

In univariable analyses, larger native lumen dimensions were associated with lower strut coverage and higher malapposition-related outcomes, whereas larger achieved stent dimensions and greater stent expansion were associated with higher strut coverage and lower malapposition-related outcomes (Table 4). Stent volume showed a weak positive association with strut coverage in univariable analysis but an inverse association after multivariable adjustment (B = 0.05, *p* = 0.040 vs. B = −0.081, *p* = 0.007). A similar change in direction was observed for mean stent area in relation to malapposed struts. Overall, the pattern of associations remained consistent across models. Complete regression analyses for all strut-level healing outcomes are provided in the Appendix A.2 (Table A10, Table A11, Table A12, Table A13 and Table A14).

### 3.4. Composite Variable Analysis Across Strut-Level Outcomes

Multivariable models identified the exploratory mismatch variable as a consistent geometric correlate across all five predefined healing outcomes (*p* < 0.01 for all), suggesting that follow-up vessel-stent geometry is inherently linked to the quality of the neointimal response (Table 5). Detailed univariable and multivariable regression results for each strut-level endpoint are shown in the Appendix A.2 (Table A15, Table A16, Table A17, Table A18 and Table A19).

**Figure 4:**
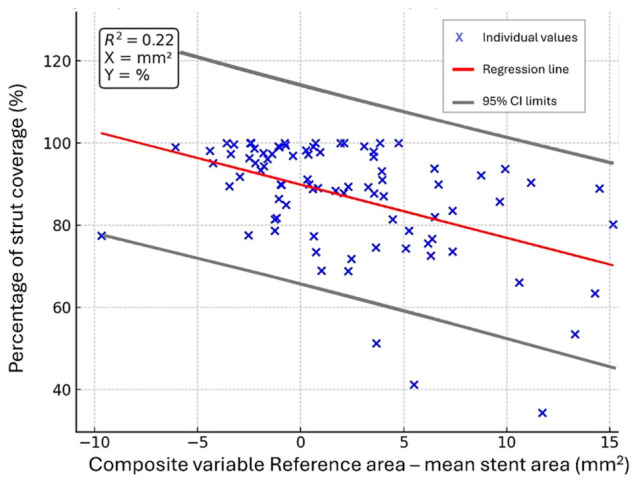
Scatter plot with linear regression showing the association between the composite variable and strut coverage.

**Figure A1:**
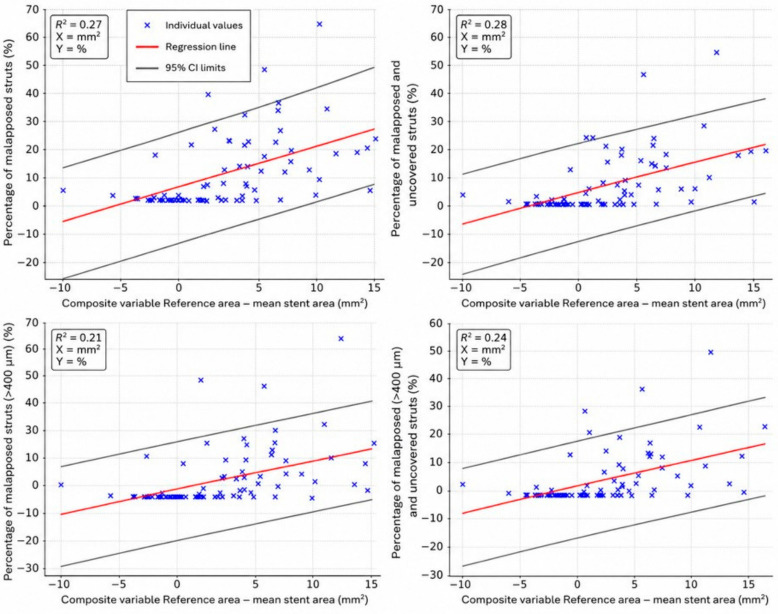
Scatter plots with linear regression showing the association between the composite variable and malapposition-related outcomes.

The inverse association between the exploratory lumen–stent mismatch variable and strut coverage is shown in Figure 4. Scatter plots for the malapposition-related outcomes are presented in the Appendix A.2 (Figure A1).

## 4. Discussion

This exploratory OCT study found that long-term healing after angiography-guided primary PCI of culprit ULM lesions was associated with the relationship between native lumen size and achieved stent area. Several observations are central. First, angiography-guided primary PCI for culprit unprotected left main lesions resulted in suboptimal stent expansion and impaired stent healing in the left main and bifurcation segments compared with the ostial and proximal LAD. Second, larger lumen dimensions at follow-up were associated with lower strut coverage and higher malapposition-related outcomes, whereas larger achieved stent area was associated with better stent healing. Importantly, reversals in the direction of associations between strut outcomes in univariable and multivariable models for stent volume (coverage) and mean stent area (malapposition) suggest that, in larger vessels, stent expansion may remain inadequate relative to native vessel dimensions despite larger absolute stent size. This observation represents the central mechanistic finding of the study and supports lumen–stent mismatch as a potential contributor to impaired vascular healing after primary PCI of culprit unprotected left main lesions. Third, the exploratory lumen–stent mismatch variable remained associated with all five predefined healing outcomes in multivariable models. Together, these findings support the hypothesis that impaired healing in the ULM region is associated with geometric mismatch between native vessel size and achieved stent area.

In contemporary DES studies, strut coverage is usually almost complete within the first months after implantation [3,15]. In contrast, the present analysis showed lower strut coverage and more frequent malapposition in proximal ULM segments, whereas healing in distal main branch segments was closer to previously reported OCT findings. This pattern is consistent with pathological and OCT data showing that uncovered and malapposed struts cluster in proximal LM and bifurcation regions [4,5,16]. In our cohort, these abnormalities were present despite the absence of angiographic restenosis or obvious clinical failure at the time of follow-up imaging.

This interpretation is biologically plausible in proximal left main bifurcation anatomy, where vessel caliber is large, lumen geometry is frequently eccentric, and local flow conditions are complex [17,18,19,20]. Prior studies have also shown that lumen enlargement and stent undersizing can contribute to malapposition [19]. Our results support the same general concept in the setting of culprit ULM PCI.

In the present study, significantly malapposed struts (>400 μm) were more often uncovered than those with smaller separation from the vessel wall, which is consistent with prior OCT and pathological data showing that larger malapposition is less likely to resolve and more likely to remain uncovered over time [11,12,15,21,22,23]. Although baseline OCT was not available and causality cannot be established, these findings support the view that larger strut-to-wall separation is one component of impaired long-term healing in proximal ULM segments.

Despite angiography-guided stent optimization, including proximal optimization techniques, favorable acute angiographic results did not consistently translate into optimal long-term strut-level healing in our cohort. This observation is concordant with prior intravascular imaging studies demonstrating the limited ability of angiography alone to accurately assess vessel size and stent expansion in large-caliber coronary segments [24]. Further, the GUIDE-DES study demonstrated that QCA-guided PCI can approximate IVUS-guided outcomes, but only when specific correction algorithms are applied and predefined sizing assumptions are respected [25]. The present findings therefore provide a mechanistic rationale for intravascular imaging-guided sizing and optimization strategies in culprit ULM PCI, particularly in large-caliber proximal bifurcation segments where angiographic assessment may underestimate vessel size or achieved underexpansion.

The clinical implications of these OCT findings should be interpreted with caution. Prior registries and meta-analyses have shown mixed results regarding the prognostic significance of malapposition overall [6,23,26,27,28,29]. The prognostic significance of malapposition may depend on its severity, timing (acute vs. late-acquired), stent generation, and coexisting features such as neoatherosclerosis or bifurcation-specific flow disturbances [28,30,31]. Although malapposition per se has not been consistently associated with adverse outcomes, larger malapposition burden may be more clinically relevant than minor malapposition [22,26,32]. Within the limitations inherent to the small sample size and exploratory study design, the present analysis was not sufficiently powered to evaluate the relationship between the observed OCT findings and corresponding clinical outcomes; therefore, the results should be interpreted as hypothesis-generating. Accordingly, the exploratory lumen–stent mismatch variable should not be regarded as a validated risk marker, procedural target, or treatment threshold, but rather as a study-specific descriptor of the geometric relationship between native vessel size and achieved stent area at follow-up. Although clinical follow-up data were available, the study was not designed to establish clinically meaningful associations between OCT-derived healing patterns and adverse clinical events.

Future studies should validate these findings in larger, prospective cohorts with systematic baseline and follow-up OCT imaging to distinguish acute from late-acquired malapposition. Standardized follow-up intervals and integration with clinical outcomes are needed to define the prognostic significance of lumen–stent mismatch.

### Study Limitations

Several limitations should be acknowledged. This was a retrospective, single-center exploratory study with a relatively small sample size, and the findings should therefore be interpreted as hypothesis-generating. The limited number of patients, together with the number of tested associations, increases the risk of model instability and type I error, in the absence of correction for multiple comparisons. The cohort consisted exclusively of hospital survivors who underwent long-term OCT follow-up and is therefore subject to survivorship and selection bias. Patients with early fatal outcomes were not represented, and the findings cannot be extrapolated to the entire population of patients with culprit unprotected left main lesions. This selection may have led to an underestimation of adverse healing patterns. Follow-up duration was variable across patients; however, follow-up < 12 months was an exclusion criterion to ensure assessment beyond the expected completion of vascular healing after drug-eluting stent implantation. Although no significant association between follow-up duration and healing outcomes was observed, the study may have been underpowered to detect such effects. Baseline OCT imaging was not available, precluding differentiation between persistent acute and late-acquired malapposition. Finally, the study was not designed or powered to assess associations between OCT-derived healing patterns and clinical outcomes, and the findings should therefore be interpreted as mechanistic rather than prognostic.

## 5. Conclusions

In this exploratory OCT study of culprit unprotected left main lesions treated with angiography-guided primary PCI, long-term healing patterns were associated with the relation between native lumen size and achieved stent area. Proximal bifurcation segments showed lower strut coverage and greater malapposition-related abnormalities than the distal main branch, and these findings were associated with larger lumen dimensions relative to achieved stent area. These findings provide a mechanistic rationale for further evaluation of intravascular imaging–guided stent optimization in left main PCI but should be interpreted in the context of the exploratory design and study limitations.

## Figures and Tables

**Table 1:** Baseline clinical, angiographic, and procedural characteristics.

Patients, n (%)	30 (100)
Age, years (mean ± SD)	57.8 ± 9.1
Male sex, n (%)	22 (73)
STEMI presentation, n (%)	20 (67)
Killip class 3–4 at admission, n (%)	2 (6)
Isolated ULMCA disease, n (%)	12 (39)
True bifurcation/trifurcation, n (%)	6 (20)/2 (7)
Pre-PCI TIMI 0–1 flow MB, n (%)	12 (40)
One-stent/two-stent technique, n (%)	27 (90)/3 (10)
Proximal stent optimization (POT), n (%)	24 (79)
Final TIMI 3 flow (all branches), n (%)	30 (100)
Angiographic success, n (%)	29 (97)
Post-PCI LVEF, % (mean ± SD)	49.0 ± 12.5

LVEF—left ventricular ejection fraction; MB—main branch; PCI—percutaneous coronary intervention; POT—proximal optimization technique; STEMI—ST-segment elevation myocardial infarction; TIMI—Thrombolysis in Myocardial Infarction; ULMCA—unprotected left main coronary artery.

**Table 2:** OCT strut-level analysis coverage and malapposition outcomes according to left-main bifurcation segments.

	LM (N = 28)	POC (N = 29)	dMB (N = 29)
Total length of analysed segment, mm (mean ± SD)	6.7 ± 4.4	2.4 ± 0.9	13.8 ± 6.7
Total number of analysed struts, n (mean ± SD)	274.8 ± 227.1	94.4 ± 43.2	733.9 ± 543.4
Total covered struts, % (mean ± SD)	82.8 ± 17.3	84.2 ± 12.4	93.9 ± 6.1
All malapposed struts, % (mean ± SD)	17.4 ± 12.5	14.2 ± 10.9	0.4 ± 0.9
Malapposed and uncovered struts, % (mean ± SD)	8.9 ± 14.2	9.2 ± 8.4	0.2 ± 0.7
Malapposed struts > 400 μm, % (mean ± SD)	7.4 ± 2.6	10.1 ± 9.4	0.0 ± 0.1
Malapposed struts > 400 μm and uncovered, % (mean ± SD)	5.6 ± 9.7	6.7 ± 6.8	0.0 ± 0.1

LM—eft main; POC, polygon of confluence; dMB, distal main branch; SD—standard deviation; μm, micrometers.

**Table 3:** Lumen, stent and neointimal hyperplasia parameters used for regression analysis models in prediction of strut healing outcomes.

	LM (N = 28)	POC (N = 29)	dMB (N = 29)	*p*
Reference lumen diameter, mm (mean ± SD)	4.5 ± 0.8		3.2 ± 0.3	<0.001
Reference lumen area, mm^2^ (mean ± SD)	16.4 ± 4.9		7.3 ± 2.0	<0.001
Minimal lumen area (MLA), mm^2^ (mean ± SD)	11.0 ± 4.9	10.9 ± 3.6	6.1 ± 2.0	<0.001
Mean lumen area (overall), mm^2^ (mean ± SD)	13.5 ± 4.9	14.0 ± 3.9	7.9 ± 1.7	0.001
Percent lumen stenosis, % (mean ± SD)	17.6 ± 20.4	9.9 ± 14.9	38.3 ± 22.7	<0.001
Minimal stent area (MSA), mm^2^ (mean ± SD)	11.5 ± 3.3	9.8 ± 2.5	7.9 ± 1.7	<0.001
Mean stent area, mm^2^ (mean ± SD)	13.9 ± 3.5	12.6 ± 3.3	9.6 ± 1.8	<0.001
Minimal stent expansion, % (mean ± SD)	72.4 ± 33.2	59.7 ± 16.6	100.5 ± 18.6	<0.001
Mean stent expansion, % (mean ± SD)	87.5 ± 35.7	75.8 ± 19.1	123.9 ± 28.3	<0.001
Total in-stent lumen volume, mm^3^ (mean ± SD)	80.7 ± 45.8	33.5 ± 17.1	109.9 ± 61.2	<0.001
Total stent volume, mm^3^ (mean ± SD)	84.1 ± 48.1	28.1 ± 13.4	131.0 ± 72.3	<0.001
Total NIH volume, mm^3^ (mean ± SD)	12.5 ± 11.5	3.6 ± 1.9	24.9 ± 17.3	<0.001
Total malapposition volume, mm^3^ (mean ± SD)	5.7 ± 8.9	7.3 ± 7.0	0.8 ± 1.0	<0.001

MLA—minimal lumen area; MSA—minimal stent area; NIH—neointimal hyperplasia; SD—standard deviation.

**Table 4:** Multivariable regression analysis of OCT-based parameters and strut healing outcomes (significant variables only).

	Univariable Regression				Multivariable Regression	
OCT Parameter	R	R^2^	B (95% CI)	*p*	B (95% CI)	*p*
**Percentage of covered struts**						
Reference area (mm^2^)	0.549	0.302	−1.19 (−1.58, −0.79)	0.000	−2.277 (−4.123, −0.43)	0.016
Stent volume (mm^3^)	0.223	0.050	0.05 (0.002, 0.09)	0.040	−0.081 (−0.139, −0.022)	0.007
NIH volume (mm^3^)	0.423	0.179	0.38 (0.205, 0.565)	0.000	0.412 (0.117, 0.707)	0.007
**Percentage of malapposed struts**						
Minimal lumen area (mm^2^)	0.632	0.399	1.96 (1.43, 2.48)	0.000	1.696 (0.402, 2.989)	0.011
Mean lumen area (mm^2^)	0.689	0.475	1.98 (1.52, 2.43)	0.000	2.062 (0.584, 3.54)	0.007
Mean stent area (mm^2^)	0.326	0.106	1.27 (0.46, 2.01)	0.002	−3.46 (−5.313, −1.606)	0.000
**Percentage of malapposed and uncovered struts**						
Mean lumen area (mm^2^)	0.704	0.495	1.502 (1.171, 1.833)	0.001	1.674 (0.628, 2.721)	0.002
Mean stent area (mm^2^)	0.335	0.112	0.970 (0.37, 1.7)	0.002	−2.983 (−4.295, −1.671)	0.000
NIH volume (mm^3^)	0.339	0.115	−0.226 (−0.360, −0.09)	0.002	0.18 (0.013, 0.347)	0.035
Malapposition volume (mm^3^)	0.715	0.511	1.004 (0.79, 1.22)	0.001	0.259 (0.003, 0.515)	0.047
**Percentage of malapposed struts > 400 μm**						
Minimal lumen area (mm^2^)	0.623	0.388	1.065 (0.773, 1.358)	0.000	0.85 (0.112, 1.587)	0.025
Mean lumen area (mm^2^)	0.679	0.461	1.076 (0.822, 1.330)	0.000	1.426 (0.584, 2.269)	0.001
Mean stent area (mm^2^)	0.320	0.103	0.689 (0.244, 1.134)	0.003	−2.455 (−3.512, −1.399)	0.000
**Percentage of malapposed (>400 μm) and uncovered struts**						
Minimal lumen area (mm^2^)	0.623	0.388	1.065 (0.773, 1.358)	0.000	0.85 (0.112, 1.587)	0.025
Mean lumen area (mm^2^)	0.679	0.461	1.076 (0.822, 1.330)	0.000	1.426 (0.584, 2.269)	0.001
Mean stent area (mm^2^)	0.320	0.103	0.689 (0.244, 1.134)	0.003	−2.455 (−3.512, −1.399)	0.000

NIH—Neointimal hyperplasia volume; OCT—optical coherence tomography.

**Table 5:** Regression analysis of composite OCT variable on strut outcomes.

	Univariable Regression				Multivariable Regression	
Strut-Healing Outcomes	R	R^2^	B (95% CI)	*p*	B (95% CI)	*p*
Percentage of covered struts	0.489	0.220	−0.170 (−0.240, −0.100)	<0.000	−2.074 (−3.595, −0.554)	0.008
Percentage of all malapposed struts	0.518	0.268	0.190 (0.122, 0.259)	<0.000	1.923 (0.666, 3.18)	0.003
Percentage of malapposed and uncovered struts	0.533	0.284	0.255 (0.166, 0.343)	<0.000	2.117 (1.065, 3.168)	0.000
Percentage of malapposed struts > 400 μm	0.459	0.210	0.226 (0.131, 0.322)	<0.000	1.286 (0.357, 2.214)	0.007
Percentage of malapposed (>400 μm) and uncovered struts	0.491	0.241	0.326 (0.199, 0.452)	<0.000	1.418 (0.693, 2.143)	0.000

µm—micrometers.

## Data Availability

The data presented in this study are available on request from the corresponding author. The data are not publicly available due to privacy and ethical restrictions.

## Abbreviations

The following abbreviations are used in this manuscript:

ACS	Acute coronary syndrome
CABG	Coronary artery bypass grafting
CI	Confidence interval
CKD	Chronic kidney disease
dMB	Distal main branch
DES	Drug-eluting stent
DK-crush	Double-kissing crush
DM	Diabetes mellitus
eGFR	Estimated glomerular filtration rate
FKBI	Final kissing balloon inflation
FU	Follow-up
IQR	Interquartile range
IVUS	Intravascular ultrasound
LAD	Left anterior descending coronary artery
LM	Left main
LVEF	Left ventricular ejection fraction
MB	Main branch
MLA	Minimal lumen area
MSA	Minimal stent area
NIH	Neointimal hyperplasia
NS	Not significant
OCT	Optical coherence tomography
PCI	Percutaneous coronary intervention
POBA	Plain old balloon angioplasty
POC	Polygon of confluence
POT	Proximal optimization technique
QCA	Quantitative coronary angiography
R^2^	Coefficient of determination
SB	Side branch
SD	Standard deviation
STEMI	ST-segment elevation myocardial infarction
TAP	T and protrusion technique
TIMI	Thrombolysis in Myocardial Infarction
TLR	Target lesion revascularization
ULM	Unprotected left main
ULMCA	Unprotected left main coronary artery

## Appendix A

### Appendix A.1. Optical Coherence Tomography

#### Appendix A.1.1. Imaging and Analysis

Following repeat coronary angiography, invasive OCT imaging was performed in 30 patients according to a standardized protocol. A 0.014-inch guidewire was positioned distal to the target segment, and an OCT catheter (Dragonfly Optis, Abbott, Santa Clara, CA, USA) was advanced at least 10 mm beyond the region of interest. Automated Z-offset calibration was performed prior to image acquisition. Automated pullback was conducted at 20 mm/s with image acquisition at 0.2 mm intervals. Imaging was performed after selective intracoronary administration of nitroglycerin (50–200 μg). Blood clearance was achieved using manual or automated injection of iso- or hypo-osmolar contrast. OCT pullback extended from the left anterior descending artery to the ostial segment of the unprotected left main coronary artery. Images were acquired and stored digitally using a commercially available OCT system (C7-XR, Abbott).

OCT recordings were reviewed to confirm intraluminal guidewire and catheter positioning and adequate image quality, including complete visualization of the stented segment, absence of major artifacts, and effective blood clearance. In cases of extraluminal positioning or inadequate image quality, guidewire repositioning and repeat OCT acquisition were performed. In patients treated with a two-stent technique for unprotected left main bifurcation lesions, side-branch OCT imaging was performed for intraprocedural assessment but was not included in the analysis.

Procedural feasibility variables included OCT image quality, contrast volume, feasibility of intraluminal catheter positioning, visualization of the ostial left main segment, and the length of stent segments excluded from analysis due to inadequate image quality.

Offline OCT analyses were performed using dedicated software (CASS Intravascular, service pack 2.1; PIE Medical, Maastricht, The Netherlands). Quantitative and qualitative analyses of the lumen, stent, and struts were conducted at 0.2 mm intervals along the entire stented segment. Cross-sections with incomplete stent visualization, residual blood artifacts, or technical limitations were excluded. Struts with indistinct contours were excluded from qualitative coverage assessment.

Strut-level classifications demonstrated excellent inter-observer reproducibility (Cohen’s kappa 0.89 for coverage and 0.92 for apposition) and intra-observer reproducibility (kappa 0.91 and 0.94, respectively), assessed on a random 10% subset of the dataset.

#### Appendix A.1.2. OCT Definitions and Variables

Strut-level analysis was performed to assess stent coverage, malapposition, expansion, and neointimal response. Struts were classified as covered only when completely covered by neointimal tissue. Partially covered struts were classified as uncovered. Strut coverage was quantified as the percentage of covered struts relative to the total number of identified struts. Neointimal thickness was measured as the distance between the luminal boundary and the luminal surface of the strut. Strut malapposition was defined as a strut-to-vessel wall distance exceeding the combined thickness of the strut and polymer. Malapposition distances > 400 μm were classified as significant. Malapposed struts were further classified as covered or uncovered. Neointimal hyperplasia (NIH) was quantified volumetrically from in-stent measurements. Percentage NIH was expressed as the ratio between minimal lumen area and reference lumen area for the corresponding segment. Mean NIH was calculated as the average NIH area across all analyzed cross-sections. Stent expansion was assessed using minimal stent area and mean stent area. Relative stent expansion was calculated as the ratio of stent area to reference lumen area for each subsegment. Malapposition was quantified as the proportion of malapposed struts relative to all identified struts and as malapposition volume between the abluminal stent contour and the vessel wall. Intraluminal thrombus was defined as an intraluminal mass protruding ≥200 μm into the lumen and was classified as white, red, or organized based on optical characteristics. Side-branch ostial coverage was defined as the ratio of the maximal length of continuous neointimal tissue across stent struts to the total length of the side-branch ostium. Neoatherosclerosis was defined as fibroatheroma or fibrocalcific plaque within the neointima with longitudinal extension >1.0 mm.

**Table A1:** Patient baseline and procedural characteristics according to angiographic and OCT follow-up status.

n/N of Patients (%)	Angiographic and OCT Follow-Up	Refused Angiographic and OCT Follow-Up	*p*
	30/209 (14.4)	27/209 (12.9)	
Age, years (mean ± SD)	57.8 ± 9.1	71.2 ± 11.2	<0.001
Male sex, n (%)	22 (73.3)	20 (74.1)	0.595
History of DM, n (%)	7 (23.3)	5 (18.5)	0.454
Cardiogenic shock at admission, n (%)	2 (6.7)	3 (11.1)	0.449
Anemia at admission, n (%)	2 (6.7)	7 (25.9)	0.070
eGFR < 60 mL/min/1.73 m^2^, n (%)	4 (13.3)	11 (40.7)	0.033
In-hospital cardiogenic shock, n (%)	4 (13.3)	4 (14.8)	0.585
Bifurcation success, n (%)	30 (100)	20 (74.1)	0.003
POT performed, n (%)	24 (80.0)	20 (76.9)	1.000
Main branch stent diameter, mm (mean ± SD)	3.5 ± 0.4	3.5 ± 0.5	0.692

DM—diabetes mellitus; eGFR—estimated glomerular filtration rate; POT—proximal optimization technique.

**Table A2:** Baseline demographic, clinical, and angiographic characteristics of the OCT population.

Characteristic	Value
Number of patients, n (%)	30 (100.0)
Age, years (mean ± SD)	57.8 ± 9.1
Male sex, n (%)	22 (73)
STEMI, n (%)	20 (67)
Killip class at admission, n (%)	
1–2	28 (94)
3–4	2 (6)
Number of diseased vessels, n (%)	
Isolated ULMCA disease	12 (39)
ULMCA + three-vessel disease	4 (13)
“MEDINA” classification, n (%)	
True bifurcation	6 (20)
Trifurcation	2 (7)
Pre-primary PCI TIMI flow 0–1, n (%)	
LM	3 (10)
dMB	9 (30)
SB	4 (13)

dMB—distal main branch; ULMCA—unprotected left main coronary artery; LM—left main; PCI—percutaneous coronary intervention; SB—side branch; STEMI—ST-segment elevation myocardial infarction; TIMI—Thrombolysis In Myocardial Infarction flow grade.

**Table A3:** Procedural and post-PCI characteristics of the OCT population.

Characteristic	Value
Number of patients, n (%)	30 (100.0)
IVUS during primary PCI, n (%)	1 (3)
Mechanical circulatory support, n (%)	0 (0.0)
PCI technique with single-stent implantation, n (%)	27 (90)
PCI technique with two-stent implantation, n (%)	3 (10)
TAP technique, n (%)	2 (7)
DK-crush technique, n (%)	1 (3)
Stent type	
Everolimus, n (%)	16 (53)
Zotarolimus, n (%)	7 (23)
Sirolimus, n (%)	6 (20)
Biolimus, n (%)	1 (3)
Optimization techniques	
POT only, n (%)	13 (42)
FKBI only, n (%)	0 (0.0)
POT + FKBI, n (%)	11 (37)
No further optimization, n (%)	6 (20)
Final TIMI 3 flow, n (%)	
LM	30 (100)
dMB	30 (100)
SB	30 (100)
Bifurcation lesion treatment success	
Overall, n (%)	29 (97)
MB success, n (%)	30 (100.0)
SB success, n (%)	29 (97)
Post-PCI LVEF (mean ± SD)	49.0 ± 12.5
Discharge therapy	
P2Y12 inhibitor at discharge	
Clopidogrel, n (%)	6 (20)
Ticagrelor, n (%)	21 (70)
Prasugrel, n (%)	3 (10)
Statin therapy at discharge, n (%)	30 (100)
Rosuvastatin, n (%)	12 (40)
Simvastatin, n (%)	12 (40)
Atorvastatin, n (%)	6 (20)

dMB—distal main branch—left ventricular ejection fraction; FKBI—final kissing balloon inflation; IVUS—intravascular ultrasound; LM—left main; MB—main branch; PCI—percutaneous coronary intervention; POT—proximal optimization technique; SB—side branch; TIMI—Thrombolysis In Myocardial Infarction flow grade.

**Table A4:** Feasibility and quality of OCT imaging.

Characteristic	Value
Number of patients, n (%)	30 (100)
OCT image quality, n (%)	
Good	29 (96.7)
Moderate	1 (3.3)
Poor	0 (0.0)
Difficulty with selective guide catheter engagement (due to stent protrusion into the ULMCA), n (%)	4 (13.2)
OCT catheter/coronary guidewire positioned beneath the stent, n (%)	6 (20.0)
Adequate visualization of the ULMCA ostium, n (%)	16 (53.3)
Stent protrusion into the aorta, n (%)	8 (26.7)
Degree of protrusion, n (%)	
Significant	4 (13.3)
Non-significant	4 (13.3)
Stent malapposition in the ostial ULMCA segment, n (%)	11 (36.7)
Location of ULMCA segment with poor visualization, n (%)	
Ostial	9 (30.0)
Mid	0 (0.0)
Distal	0 (0.0)
Length of ULMCA segment with poor visualization, mm (mean ± SD)	1.83 ± 0.7
Location of stented segment with poor visualization, n (%)	
Ostial	9 (30.0)
Mid	0 (0.0)
Distal	0 (0.0)
Length of stented segment with poor visualization, mm (mean ± SD)	1.68 ± 0.6

**Table A5:** Qualitative OCT analysis.

Characteristic	Value
Number of patients, n (%)	30 (100.0)
Stent deformation, n (%)	3 (10.0)
Location of stent deformation, n (%)	
Ostial	2 (6.7)
Mid	1 (3.3)
Distal	0 (0.0)
Type of stent deformation, n (%)	
Longitudinal compression	2 (6.7)
Abluminal deformation	1 (3.3)
Presence of “floating” stent struts across the side-branch ostium, n (%)	25 (83.3)
Neointimal proliferation over “floating” struts, n (%)	18 (60.0)
Percentage of side-branch ostial obstruction by proliferation, % (mean ± SD)	58.6 ± 21.4
Thrombus, n (%)	4 (13.3)
Thrombus location, n (%)	
Neocarina	2 (6.7)
Floating struts	1 (3.3)
LAD ostium	1 (3.3)
Type of thrombus, n (%)	
Organized	3 (10.0)
White	1 (3.3)
Red	0 (0.0)
In-stent neoatherosclerosis, n (%)	10 (33.3)
Presence of calcium deposits within the stent, n (%)	3 (10.0)
Unhealed dissection, n (%)	0 (0.0)

SD—standard deviation; LAD—left anterior descending coronary artery.

**Table A6:** Tabular overview of reinterventions during repeat coronary angiography and OCT follow-up.

Characteristic	Value
Number of patients, n (%)	30 (100.0)
Repeat PCI, n (%)	17 (56.7)
TLR only	8 (26.7)
Non-TLR only	5 (16.5)
TLR + non-TLR	4 (13.3)
Reason for TLR	
MB restenosis	1 (3.3)
SB restenosis	3 (10.0)
Malapposition	4 (13.3)
MB restenosis + malapposition	2 (6.7)
MB restenosis + SB restenosis	2 (6.7)
POBA only, n (%)	9 (30.0)
New stent implantation, n (%)	4 (13.3)
POBA only in the ULMCA, n (%)	6 (20.0)
New stent implantation in the ULMCA, n (%)	2 (6.7)
Procedural success of repeat PCI	16 (94.1%) overall; 11 (91.7%) in the ULMCA

ULMCA—unprotected left main coronary artery; MB—main branch; non-TLR—non–target lesion revascularization; PCI—percutaneous coronary intervention; POBA—plain old balloon angioplasty; SB—side branch; TLR—target lesion revascularization.

**Table A7:** OCT-derived strut-level coverage and malapposition outcomes according to left-main bifurcation sub-segments.

	All Segments (N = 30)	LM (N = 28)	POC (N = 29)	dMB (N = 29)	*p*
Total length of analysed segment, mm (mean ± SD)	21.9 ± 6.4	6.7 ± 4.4	2.4 ± 0.9	13.8 ± 6.7	# < 0.001; $ < 0.001; £ 0.002; € < 0.001
Total number of analyzed struts, n (mean ± SD)	1056.8 ± 570.3	274.8 ± 227.1	94.4 ± 43.2	733.9 ± 543.4	# < 0.001; $ < 0.001; £ 0.003; € < 0.001
Number of analyzed struts per cross-section, n (mean ± SD)	9.8 ± 2.1	8.7 ± 2.6	9.3 ± 2.2	10.8 ± 2.6	# 0.460
Total covered struts (covered + malapposed but covered), % (mean ± SD)	90.7 ± 6.6	82.8 ± 17.3	84.2 ± 12.4	93.9 ± 6.1	# 0.003; $ 0.876; £ 0.009; € 0.001
Total uncovered struts (uncovered + malapposed and uncovered), % (mean ± SD)	9.3 ± 6.6	17.2 ± 17.3	15.8 ± 12.4	6.0 ± 6.0	# 0.003; $ 0.876; £ 0.009; € 0.001
All malapposed struts, % (mean ± SD)	4.2 ± 3.9	17.4 ± 12.5	14.2 ± 10.9	0.4 ± 0.9	# < 0.001; $ 0.130; £ < 0.001; € < 0.001
Malapposed and uncovered struts (all), % (mean ± SD)	2.8 ± 2.7	8.9 ± 14.2	9.2 ± 8.4	0.2 ± 0.7	# 0.003; $ 0.277; £ < 0.001; € < 0.001
Malapposed struts ≤ 400 μm, % (mean ± SD)	1.8 ± 2.1	4.7 ± 6.4	4.1 ± 3.9	0.3 ± 0.8	# < 0.001; $ 0.881; £ 0.003; € < 0.001
Malapposed struts ≤ 400 μm and uncovered, % (mean ± SD)	1.1 ± 1.6	8.7 ± 25.8	2.9 ± 4.9	1.4 ± 3.6	# 0.007; $ 0.782; £ 0.003; € 0.011
Malapposed struts > 400 μm, % (mean ± SD)	2.4 ± 2.4	7.4 ± 2.6	10.1 ± 9.4	0.0 ± 0.1	# < 0.001; $ 0.031; £ < 0.001; € < 0.001
Malapposed struts > 400 μm and uncovered, % (mean ± SD)	1.7 ± 1.8	5.6 ± 9.7	6.7 ± 6.8	0.0 ± 0.1	# < 0.001; $ 0.137; £ < 0.001; € < 0.001
Percentage of cross-sections with >30% malapposed struts, % (mean ± SD)	6.7 ± 6.5	20.6 ± 30.9	20.4 ± 19.1	0.2 ± 1.3	# < 0.001; $ 0.321; £ < 0.001; € < 0.001
Percentage of cross-sections with at least one malapposed strut, % (mean ± SD)	13.2 ± 8.7	28.8 ± 36.4	48.8 ± 30.9	2.6 ± 4.9	# < 0.001; $ 0.028; £ 0.001; € < 0.001
Maximum consecutive length with at least one malapposed strut, % (mean ± SD)	1.9 ± 1.8	1.3 ± 1.6	0.9 ± 0.9	0.2 ± 0.4	# < 0.001; $ 0.723; £ 0.003; € < 0.001
Segments with consecutive malapposition length > 1 mm, n/N (%)	18/30 (60)	11/28 (39.3)	11/29 (37.9)	1/29 (3.4)	# < 0.001; $ 1.000; £ 0.001; € 0.001

SD—standard deviation; dMB—distal main branch; LM—left main; POC—polygon of confluence; #—between all segments; $—between LM and POC; £—between LM and dMB; €—between POC and dMB.

**Table A8:** Descriptive analysis of malapposed struts according to malapposition distance.

Parameter	Malapposition ≤ 400 µm	Malapposition > 400 µm
Total number of struts, n	660	541
Mean number per patient, n (mean ± SD)	24.9 ± 22.4	20.8 ± 29.2
Minimum number per patient, n	1	1
Maximum number per patient, n	86	149
Interquartile range (IQR)	25.0	21.5
Median, n	18.0	12.5
Total number of uncovered struts, n	369	475
Mean number of uncovered struts per patient, n (mean ± SD)	17.9 ± 16.1	14.2 ± 27.3
Percentage of uncovered struts, % (mean ± SD)	57.7 ± 39.6	73.4 ± 29.2

SD—standard deviation.

**Table A9:** Quantitative luminal and volumetric OCT measurements according to ULMCA bifurcation segments.

	All Segments (N = 30)	LM (N = 28)	POC (N = 29)	dMB (N = 29)	*p*
Number of analyzed cross-sections, n (mean ± SD)	109.4 ± 32.0	33.4 ± 21.9	11.8 ± 4.4	68.8 ± 33.3	#& < 0.001
Length of analyzed stented segment, cm (mean ± SD)	21.8 ± 6.4	6.6 ± 4.4	2.4 ± 0.9	13.7 ± 6.7	#& < 0.001
Reference lumen area, mm^2^ (mean ± SD)	NA	16.4 ± 4.9		7.3 ± 2.0	#£€ < 0.001
Minimum lumen diameter at reference area, mm (mean ± SD)	NA	4.1 ± 0.8		2.9 ± 0.4	# < 0.001
Maximum lumen diameter at reference area, mm (mean ± SD)	NA	5.1 ± 0.9		3.4 ± 0.2	# < 0.001
Lumen eccentricity at reference area, n	0.2 ± 0.1	0.2 ± 0.1		0.2 ± 0.1	#£€ NS
MLA, mm^2^ (mean ± SD)	5.8 ± 2.1	11.0 ± 4.9	10.9 ± 3.6	6.1 ± 2.0	#£€ < 0.001; $ 0.774
Minimum lumen diameter at MLA, mm (mean ± SD)	2.4 ± 0.5	3.1 ± 0.8	2.9 ± 0.5	2.5 ± 0.5	# 0.001; $ 0.486; £ 0.002; € 0.001
Maximum lumen diameter at MLA, mm (mean ± SD)	2.9 ± 0.6	4.2 ± 0.9	4.5 ± 1.1	3.0 ± 0.5	# 0.001; $ 0.651; £ < 0.001; € < 0.001
Lumen eccentricity at MLA, n	0.2 ± 0.1	0.2 ± 0.1	0.3 ± 0.2	0.2 ± 0.1	#& NS
Mean lumen area (all), mm^2^ (mean ± SD)	9.8 ± 2.1	13.5 ± 4.9	14.0 ± 3.9	7.9 ± 1.7	# 0.001; $ 0.394; £ < 0.001; € < 0.001
Mean minimum lumen diameter (all), mm (mean ± SD)	3.1 ± 0.4	3.6 ± 0.7	3.0 ± 0.5	2.9 ± 0.4	# 0.001; $ 0.001; £ < 0.001; € 0.355
Mean maximum lumen diameter (all), mm (mean ± SD)	3.9 ± 0.4	4.6 ± 0.9	5.4 ± 0.9	3.4 ± 0.4	#& < 0.001
Lumen area eccentricity (all), n	0.2 ± 0.1	0.2 ± 0.1	0.4 ± 0.1	0.2 ± 0.1	# <0.001; $ < 0.001; £ 0.015; € < 0.001
Minimum lumen diameter, mm (mean ± SD)	2.1 ± 0.5	3.1 ± 0.7	2.5 ± 0.5	2.4 ± 0.5	# < 0.001; $ 0.002; £ 0.002; € 0.994
Lumen stenosis, % (mean ± SD)	NA	17.6 ± 20.4	9.9 ± 14.9	38.3 ± 22.7	# < 0.001; $ 0.125; £ 0.001; € < 0.001
Total in-stent lumen volume, mm^3^ (mean ± SD)	213.5 ± 75.6	80.7 ± 45.8	33.5 ± 17.1	109.9 ± 61.2	# <0.001; $ < 0.001; £ 0.064; € < 0.001
Total stent volume, mm^3^ (mean ± SD)	232.7 ± 75.9	84.1 ± 48.1	28.1 ± 13.4	131.0 ± 72.3	# < 0.001; $ < 0.001; £ 0.013; € < 0.001
Total NIH volume, mm^3^ (mean ± SD)	39.4 ± 17.7	12.5 ± 11.5	3.6 ± 1.9	24.9 ± 17.3	# < 0.001; $ < 0.001; £ 0.003; € < 0.001
NIH area at MLA cross-section, mm^2^ (mean ± SD)	2.8 ± 1.8	2.1 ± 1.2	1.7 ± 0.9	2.5 ± 1.7	# < 0.001; $ < 0.001; £ 0.003; € < 0.001
Mean NIH area, mm^2^ (mean ± SD)	1.8 ± 0.7	1.8 ± 1.0	1.7 ± 0.7	1.8 ± 0.8	# 0.958;
Total malapposition volume, mm^3^ (mean ± SD)	12.9 ± 12.1	5.7 ± 8.9	7.3 ± 7.0	0.8 ± 1.0	# < 0.001; $ 0.065; £ 0.004; € < 0.001
Malapposition area per cross-section, mm^2^ (mean ± SD)	0.6 ± 0.5	1.7 ± 2.8	2.9 ± 2.6	0.3 ± 0.4	# < 0.001; $ 0.010; £ 0.001; € < 0.001
Mean stent area, mm^2^ (mean ± SD)	11.0 ± 1.9	13.9 ± 3.5	12.6 ± 3.3	9.6 ± 1.8	# < 0.001; $ 0.095; £ < 0.001; € < 0.001
Stent area at MLA cross-section, mm^2^ (mean ± SD)	8.8 ± 2.0	12.3 ± 3.5	11.8 ± 2.9	8.7 ± 1.9	# < 0.001; $ 1.000; £ < 0.001; € < 0.001
Minimum stent area (MSA), mm^2^ (mean ± SD)	7.9 ± 1.9	11.5 ± 3.3	9.8 ± 2.5	7.9 ± 1.7	# < 0.001; $ 0.058; £ < 0.001; € 0.016
Minimum stent expansion, % (mean ± SD)	NA	72.4 ± 33.2	59.7 ± 16.6	100.5 ± 18.6	# < 0.001; $ 0.150; £ < 0.001; € < 0.001
Mean stent expansion, % (mean ± SD)	NA	87.5 ± 35.7	75.8 ± 19.1	123.9 ± 28.3	# < 0.001; $ 0.245; £ < 0.001; € < 0.001
Difference between reference lumen area and mean stent area, mm^2^ (mean ± SD)	NA	3.46 ± 5.4	4.8 ± 4.2	−1.6 ± 1.7	# < 0.001; $ 0.207; £ < 0.001; € < 0.001

#—between all segments; $—LM vs. POC; £—LM vs. dMB; €—POC vs. dMB. MLA—minimal lumen area; MSA—minimal stent area; NIH—neointimal hyperplasia; SD—standard deviation; ULMCA—unprotected left main coronary artery; &—between segments.

### Appendix A.2. Regression Analysis

#### Appendix A.2.1. Strut Coverage

In univariable analyses, reference diameter, reference lumen area, minimal and mean lumen area were inversely associated with strut endothelial coverage, whereas stent expansion indices and neointimal hyperplasia (NIH) volume showed positive associations. Malapposition volume was negatively associated with strut coverage (Table A10). In multivariable analysis, reference lumen area, stent volume, and NIH volume remained associated with strut endothelial coverage (Table A10).

**Table A10:** Uni- and multivariate regression analysis for strut coverage.

	Univariable Regression				Multivariable Regression	
	R	R^2^	B (95% CI)	*p*	B (95% CI)	*p*
Reference diameter (mm)	0.526	0.277	−7.45 (−10.1, −4.82)	0.000	11.729 (−2.566, 26.024)	0.106
Reference area (mm^2^)	0.549	0.302	−1.19 (−1.58, −0.79)	0.000	−2.277 (−4.123, −0.43)	0.016
Minimal lumen area (mm^2^)	0.591	0.349	−1.85 (−2.41, −1.30)	0.000	−1.441 (−3.056, 0.174)	0.079
Mean lumen area (mm^2^)	0.624	0.389	−1.81 (−2.31, −1.32)	0.000	−1.608 (−3.453, 0.237)	0.087
Percent lumen stenosis (%)	0.300	0.090	0.178 (0.05, 0.30)	0.005	−0.102 (−0.25, 0.047)	0.178
Minimal stent area (mm^2^)	0.240	0.058	−1.20 (−2.07, −0.13)	0.027	1.038 (−1.32, 3.395)	0.383
Mean stent area (mm^2^)	0.332	0.110	−1.31 (−2.12, −0.49)	0.002	1.734 (−0.579, 4.048)	0.139
Minimal stent expansion (%)	0.387	0.150	0.18 (0.09, 0.27)	0.000	−0.087 (−0.421, 0.248)	0.607
Mean stent expansion (%)	0.379	0.143	0.15 (0.07, 0.23)	0.000	0.053 (−0.198, 0.303)	0.677
Lumen volume (mm^3^)	0.081	0.007	0.02 (−0.03, 0.07)	0.459		
Stent volume (mm^3^)	0.223	0.050	0.05 (0.002, 0.09)	0.040	−0.081 (−0.139, −0.022)	0.007
NIH volume (mm^3^)	0.423	0.179	0.38 (0.205, 0.565)	0.000	0.412 (0.117, 0.707)	0.007
Malapposition volume (mm^3^)	0.573	0.328	−1.09 (−1.44, −0.75)	0.000	−0.232 (−0.683, 0.22)	0.310

#### Appendix A.2.2. Strut Malapposition

Univariable analyses showed positive associations between strut malapposition and reference diameter, reference area, and minimal and mean lumen area. Mean stent area and stent expansion indices demonstrated inverse associations (Table A11). In multivariable analysis, minimal and mean lumen area remained associated with strut malapposition, while mean stent area showed an independent inverse association (Table A11).

**Table A11:** Uni- and multivariate regression analysis for strut malapposition.

	Univariable Regression				Multivariable Regression	
	R	R^2^	B (95% CI)	*p*	B (95% CI)	*p*
Reference diameter (mm)	0.588	0.346	8.22 (5.75, 10.68)	0.000	−2.005 (−13.457, 9.447)	0.728
Reference area (mm^2^)	0.584	0.341	1.25 (0.87, 1.63)	0.000	1.003 (−0.477, 2.482)	0.181
Minimal lumen area (mm^2^)	0.632	0.399	1.96 (1.43, 2.48)	0.000	1.696 (0.402, 2.989)	0.011
Mean lumen area (mm^2^)	0.689	0.475	1.98 (1.52, 2.43)	0.000	2.062 (0.584, 3.54)	0.007
Percent lumen stenosis (%)	0.302	0.091	−0.18 (−0.29, −0.56)	0.005	0.082 (−0.037, 0.201)	0.175
Minimal stent area (mm^2^)	0.257	0.091	1.16 (0.21, 2.11)	0.018	0.099 (−1.789, 1.988)	0.917
Mean stent area (mm^2^)	0.326	0.106	1.27 (0.46, 2.01)	0.002	−3.46 (−5.313, −1.606)	0.000
Minimal stent expansion (%)	0.435	0.189	−0.19 (−0.29, −0.11)	0.000	−0.083 (−0.351, 0.185)	0.538
Mean stent expansion (%)	0.426	0.182	−0.16 (−0.24, −0.09)	0.000	0.104 (−0.097, 0.304)	0.307
Lumen volume (mm^3^)	0.199	0.040	−0.05 (−0.10, 0.04)	0.067		
Stent volume (mm^3^)	0.317	0.101	−0.06 (−0.11, −0.02)	0.003	−0.024 (−0.07, 0.023)	0.320
NIH volume (mm^3^)	0.367	0.134	−0.33 (−0.51, −0.15)	0.001	0.152 (−0.085, 0.388)	0.205
Malapposition volume (mm^3^)	0.715	0.511	1.35 (1.06, 1.64)	0.000	0.349 (−0.013, 0.71)	0.059

NIH—Neointimal hyperplasia.

#### Appendix A.2.3. Composite Outcome of Malapposed and Uncovered Struts

In univariable analyses, reference lumen area and minimal and mean lumen area were positively associated with the composite outcome of malapposed and uncovered struts, whereas mean stent area and stent expansion indices showed inverse associations (Table A12). In multivariable analysis, reference lumen area, minimal lumen area, and mean lumen area remained associated with this composite outcome, while mean stent area retained an independent inverse association (Table A12).

**Table A12:** Uni- and multivariate regression analysis for malapposition with strut uncoverage.

	Univariable Regression				Multivariable Regression	
	R	R^2^	B (95% CI)	*p*	B (95% CI)	*p*
Reference diameter (mm)	0.580	0.336	6.22 (4.313, 8.13)	<0.001	−5.544 (−15.37, 4.288)	0.265
Reference area (mm^2^)	0.593	0.351	0.971 (0.683, 1.259)	<0.001	1.771 (0.501, 3.041)	0.007
Minimal lumen area (mm^2^)	0.609	0.371	1.448 (1.037, 1.860)	<0.001	1.296 (0.186, 2.407)	0.023
Mean lumen area (mm^2^)	0.654	0.428	1.440 (1.077, 1.804)	<0.001	1.754 (0.485, 3.024)	0.007
Percent lumen stenosis (%)	0.279	0.078	−0.247 (−0.22, −0.031)	0.010	0.071 (−0.031, 0.174)	0.169
Minimal stent area (mm^2^)	0.244	0.06	0.848 (0.113, 1.584)	0.024	−0.529 (−2.15, 1.093)	0.518
Mean stent area (mm^2^)	0.319	0.102	0.954 (0.336, 1.573)	0.003	−2.687 (−4.279, −1.096)	0.001
Minimal stent expansion (%)	0.423	0.179	−0.148 (−0.22, −0.079)	<0.001	0.039 (−0.191, 0.269)	0.735
Mean stent expansion (%)	0.417	0.174	−0.122 (−0.18, −0.064)	<0.001	0.036 (−0.136, 0.209)	0.676
Lumen volume (mm^3^)	0.173	0.03	−0.032 (−0.072, 0.008)	0.114		
Stent volume (mm^3^)	0.285	0.081	−0.044 (−0.07, −0.012)	0.008	−0.009 (−0.05, 0.031)	0.640
NIH volume (mm^3^)	0.357	0.127	−0.246 (−0.39, −0.105)	<0.001	0.093 (−0.11, 0.296)	0.364
Malapposition volume (mm^3^)	0.640	0.409	0.927 (0.684, 1.171)	<0.001	0.053 (−0.257, 0.364)	0.734

NIH—Neointimal hyperplasia.

#### Appendix A.2.4. Significant Strut Malapposition

Univariable analyses demonstrated significant associations between significant strut malapposition and reference lumen dimensions, particularly mean lumen area. Mean stent area, malapposition volume, and NIH volume were also associated (Table A13). In multivariable analysis, minimal and mean lumen area, mean stent area, NIH volume, and malapposition volume remained associated with significant strut malapposition (Table A13).

**Table A13:** Uni- and multivariate regression analysis for significant strut malapposition.

	Univariable Regression				Multivariable Regression	
	R	R^2^	B (95% CI)	*p*	B (95% CI)	*p*
Reference diameter (mm)	0.560	0.313	5.818 (3.94, 7.70)	0.001	2.201 (−5.903, 10.305)	0.590
Reference area (mm^2^)	0.543	0.295	0.862 (0.57, 1.15)	0.001	0.269 (−0.778, 1.316)	0.610
Minimal lumen area (mm^2^)	0.646	0.417	1.488 (1.10, 1.87)	0.001	1.314 (0.398, 2.229)	0.006
Mean lumen area (mm^2^)	0.704	0.495	1.502 (1.171, 1.833)	0.001	1.674 (0.628, 2.721)	0.002
Percent lumen stenosis (%)	0.302	0.091	−0.131 (−0.22, −0.04)	0.005	0.071 (−0.013, 0.156)	0.097
Minimal stent area (mm^2^)	0.288	0.083	0.970 (0.27, 1.67)	0.008	0.363 (−0.974, 1.699)	0.590
Mean stent area (mm^2^)	0.335	0.112	0.970 (0.37, 1.7)	0.002	−2.983 (−4.295, −1.671)	0.000
Minimal stent expansion (%)	0.366	0.134	−0.125 (−0.19, −0.06)	0.001	−0.076 (−0.265, 0.114)	0.429
Mean stent expansion (%)	0.365	0.134	−0.104 (−0.16, −0.05)	0.001	0.122 (−0.02, 0.264)	0.091
Lumen volume (mm^3^)	0.205	0.042	−0.037 (−0.08, 0.00)	0.06		
Stent volume (mm^3^)	0.315	0.099	−0.047 (−0.08, −0.02)	0.003	−0.031 (−0.064, 0.002)	0.066
NIH volume (mm^3^)	0.339	0.115	−0.226 (−0.36, −0.09)	0.002	0.18 (0.013, 0.347)	0.035
Malapposition volume (mm^3^)	0.715	0.511	1.004 (0.79, 1.22)	0.001	0.259 (0.003, 0.515)	0.047

NIH—Neointimal hyperplasia.

#### Appendix A.2.5. Composite Outcome of Significant Malapposition and Uncovered Struts

Univariable analyses identified reference diameter and mean lumen area as positively associated with the composite outcome of significant malapposition and uncovered struts, while mean stent area showed an inverse association (Table A14). In multivariable analysis, mean lumen area, mean stent area, and reference diameter remained associated with this composite outcome (Table A14).

**Table A14:** Uni- and multivariate regression analysis for significant malapposition with strut uncoverage.

	Univariable Regression				Multivariable Regression	
	R	R^2^	B (95% CI)	*p*	B (95% CI)	*p*
Reference diameter (mm)	0.565	0.319	4.354 (2.965, 5.743)	0.000	−1.131 (−7.658, 5.397)	0.731
Reference area (mm^2^)	0.560	0.313	0.661 (0.447, 0.874)	0.000	0.795 (−0.049, 1.638)	0.064
Minimal lumen area (mm^2^)	0.623	0.388	1.065 (0.773, 1.358)	0.000	0.85 (0.112, 1.587)	0.025
Mean lumen area (mm^2^)	0.679	0.461	1.076 (0.822, 1.330)	0.000	1.426 (0.584, 2.269)	0.001
Percent lumen stenosis (%)	0.282	0.080	−0.091 (−0.159, −0.023)	0.009	0.058 (−0.01, 0.126)	0.091
Minimal stent area (mm^2^)	0.292	0.085	0.729 (0.207, 1.252)	0.007	0.294 (−0.783, 1.37)	0.588
Mean stent area (mm^2^)	0.320	0.103	0.689 (0.244, 1.134)	0.003	−2.455 (−3.512, −1.399)	0.000
Minimal stent expansion (%)	0.365	0.134	−0.092 (−0.144, −0.041)	0.001	−0.031 (−0.184, 0.121)	0.683
Mean stent expansion (%)	0.376	0.141	−0.079 (−0.122, −0.037)	0.000	0.087 (−0.028, 0.201)	0.135
Lumen volume (mm^3^)	0.192	0.037	−0.026 (−0.054, 0.003)	0.078		
Stent volume (mm^3^)	0.300	0.090	−0.034 (−0.057, −0.010)	0.005	−0.02 (−0.046, 0.007)	0.148
NIH volume (mm^3^)	0.339	0.115	−0.168 (−0.270, −0.066)	0.002	0.117 (−0.017, 0.252)	0.087
Malapposition volume (mm^3^)	0.685	0.469	0.715 (0.549, 0.881)	0.000	0.101 (−0.105, 0.307)	0.334

NIH—Neointimal hyperplasia.

Across all analyzed outcomes, larger lumen dimensions and smaller achieved stent dimensions were consistently associated with strut malapposition and lack of strut coverage in both univariable and multivariable models.

#### Appendix A.2.6. Regression Analysis with Composite Variable

**Table A15:** Impact of reference lumen area and mean stent area mismatch on stent strut coverage at follow-up.

	Univariate Regression				Multivariable Regression	
Variable	R	R^2^	B (95% CI)	*p*	B (95% CI)	*p*
Reference diameter (mm)	0.496	0.246	−7.02 (−9.68, −4.33)	0.000	10.117 (−1.372, 21.605)	0.083
Minimal lumen area (mm^2^)	0.591	0.349	−1.85 (−2.41, −1.30)	0.000	−1.505 (−3.077, 0.067)	0.060
Mean lumen area (mm^2^)	0.624	0.389	−1.81 (−2.31, −1.32)	0.000	−1.616 (−3.45, 0.217)	0.083
Lumen stenosis, %	0.300	0.090	0.178 (0.05, 0.30)	0.005	−0.099 (−0.247, 0.048)	0.184
Minimal stent area (mm^2^)	0.240	0.058	−1.20 (−2.07, −0.13)	0.027	0.782 (−1.184, 2.748)	0.430
Minimum stent expansion, %	0.387	0.150	0.18 (0.09, 0.27)	0.000	−0.049 (−0.324, 0.226)	0.723
Mean stent expansion, %	0.379	0.143	0.15 (0.07, 0.23)	0.000	0.02 (−0.165, 0.204)	0.832
Lumen volume (mm^3^)	0.081	0.007	0.02 (−0.03, 0.07)	0.459		
Stent volume (mm^3^)	0.223	0.050	0.05 (0.002, 0.09)	0.040	−0.083 (−0.14, −0.026)	0.005
NIH volume (mm^3^)	0.423	0.179	0.38 (0.205, 0.565)	0.000	0.41 (0.117, 0.703)	0.007
Malapposition volume (mm^3^)	0.573	0.328	−1.09 (−1.44, −0.75)	0.000	−0.215 (−0.655, 0.225)	0.334
Difference between reference lumen area and mean stent area (mm^2^)	0.489	0.220	−0.170 (−0.240, −0.100)	<0.000	−2.074 (−3.595, −0.554)	0.008

NIH—Neointimal hyperplasia.

**Table A16:** Impact of reference lumen area and mean stent area mismatch on strut being malapposed at follow-up.

	Univariate Regression				Multivariate Regression	
Variable	R	R^2^	B (95% CI)	*p*	B (95% CI)	*p*
Reference diameter (mm)	0.578	0.334	8.06 (5.58, 10.55)	0.000	−9.463 (−18.961, 0.035)	0.051
Minimal lumen area (mm^2^)	0.632	0.399	1.96 (1.43, 2.48)	0.000	1.407 (0.107, 2.707)	0.034
Mean lumen area (mm^2^)	0.689	0.475	1.98 (1.52, 2.43)	0.000	2.026 (0.51, 3.542)	0.010
Lumen stenosis, %	0.302	0.091	−0.18 (−0.29, −0.56)	0.005	0.092 (−0.03, 0.214)	0.136
Minimal stent area (mm^2^)	0.257	0.091	1.16 (0.21, 2.11)	0.018	−1.034 (−2.659, 0.591)	0.209
Minimum stent expansion, %	0.435	0.189	−0.19 (−0.29, −0.11)	0.000	0.084 (−0.143, 0.311)	0.465
Mean stent expansion, %	0.426	0.182	−0.16 (−0.24, −0.09)	0.000	−0.045 (−0.198, 0.107)	0.555
Lumen volume (mm^3^)	0.199	0.040	−0.05 (−0.10, 0.04)	0.067		
Stent volume (mm^3^)	0.317	0.101	−0.06 (−0.11, −0.02)	0.003	−0.033 (−0.08, 0.014)	0.163
NIH volume (mm^3^)	0.367	0.134	−0.33 (−0.51, −0.15)	0.001	0.143 (−0.099, 0.386)	0.242
Malapposition volume (mm^3^)	0.715	0.511	1.35 (1.06, 1.64)	0.000	0.425 (0.061, 0.789)	0.023
Difference between reference lumen area and mean stent area (mm^2^)	0.518	0.268	0.190 (0.122, 0.259)	0.518	1.923 (0.666, 3.18)	0.003

NIH—Neointimal hyperplasia.

**Table A17:** Impact of reference lumen area and mean stent area mismatch on strut being malapposed and uncovered at follow-up.

	Univariate Regression				Multivariable Regression	
Variable	R	R^2^	B (95% CI)	*p*	B (95% CI)	*p*
Reference diameter (mm)	0.565	0.319	6.054 (4.125, 7.982)	<0.001	−8.342 (−16.285, −0.399)	0.040
Minimal lumen area (mm^2^)	0.609	0.371	1.448 (1.037, 1.860)	<0.001	1.189 (0.102, 2.276)	0.033
Mean lumen area (mm^2^)	0.654	0.428	1.440 (1.077, 1.804)	<0.001	1.742 (0.474, 3.01)	0.008
Lumen stenosis, %	0.279	0.078	−0.247 (−0.219, −0.031)	0.010	0.075 (−0.027, 0.177)	0.146
Minimal stent area (mm^2^)	0.244	0.06	0.848 (0.113, 1.584)	0.024	−0.949 (−2.308, 0.41)	0.168
Minimum stent expansion, %	0.423	0.179	−0.148 (−0.218, −0.079)	<0.001	0.101 (−0.089, 0.291)	0.291
Mean stent expansion, %	0.417	0.174	−0.122 (−0.180, −0.064)	<0.001	−0.019 (−0.147, 0.108)	0.766
Lumen volume (mm^3^)	0.173	0.03	−0.032 (−0.072, 0.008)	0.114		
Stent volume (mm^3^)	0.285	0.081	−0.044 (−0.077, −0.012)	0.008	−0.013 (−0.053, 0.026)	0.510
NIH volume (mm^3^)	0.357	0.127	−0.246 (−0.386, −0.105)	<0.001	0.09 (−0.113, 0.292)	0.380
Malapposition volume (mm^3^)	0.64	0.409	0.927 (0.684, 1.171)	<0.001	0.081 (−0.223, 0.386)	0.595
Difference between reference lumen area and mean stent area (mm^2^)	0.533	0.284	0.255 (0.166, 0.343)	0.533	2.117 (1.065, 3.168)	0.000

NIH—Neointimal hyperplasia.

**Table A18:** Impact of reference lumen area and mean stent area mismatch on strut being significantly malapposed at follow-up.

	Univariate Regression				Multivariable Regression	
Variable	R	R^2^	B (95% CI)	*p*	B (95% CI)	*p*
Reference diameter (mm)	0.562	0.316	5.835 (3.96, 7.71)	0.001	−6.023 (−13.041, 0.996)	0.091
Minimal lumen area (mm^2^)	0.646	0.417	1.488 (1.10, 1.87)	0.001	0.995 (0.034, 1.955)	0.043
Mean lumen area (mm^2^)	0.704	0.495	1.502 (1.171, 1.833)	0.001	1.635 (0.515, 2.755)	0.005
Lumen stenosis, %	0.302	0.091	−0.131 (−0.22, −0.04)	0.005	0.083 (−0.007, 0.173)	0.071
Minimal stent area (mm^2^)	0.288	0.083	0.970 (0.27, 1.67)	0.008	−0.891 (−2.092, 0.31)	0.144
Minimum stent expansion, %	0.366	0.134	−0.125 (−0.19, −0.06)	0.001	0.109 (−0.059, 0.277)	0.2
Mean stent expansion, %	0.365	0.134	−0.104 (−0.16, −0.05)	0.001	−0.043 (−0.155, 0.07)	0.455
Lumen volume (mm^3^)	0.205	0.042	−0.037 (−0.08, 0.00)	0.06		
Stent volume (mm^3^)	0.315	0.099	−0.047 (−0.08, −0.02)	0.003	−0.042 (−0.077, −0.007)	0.019
NIH volume (mm^3^)	0.339	0.115	−0.226 (−0.36, −0.09)	0.002	0.171 (−0.008, 0.35)	0.061
Malapposition volume (mm^3^)	0.715	0.511	1.004 (0.79, 1.22)	0.001	0.343 (0.074, 0.612)	0.013
Difference between reference lumen area and mean stent area (mm^2^)	0.459	0.210	0.226 (0.131, 0.322)	0.459	1.286 (0.357, 2.214)	0.007

NIH—Neointimal hyperplasia.

**Table A19:** Impact of reference lumen area and mean stent area mismatch on strut being significantly malapposed and uncovered at follow-up.

	Univariate Regression				Multivariable Regression	
Variable	R	R^2^	B (95% CI)	*p*	B (95% CI)	*p*
Reference diameter (mm)	0.565	0.319	4.354 (2.965, 5.743)	0.000	−6.167 (−11.648, −0.685)	0.028
Minimal lumen area (mm^2^)	0.623	0.388	1.065 (0.773, 1.358)	0.000	0.654 (−0.096, 1.405)	0.086
Mean lumen area (mm^2^)	0.679	0.461	1.076 (0.822, 1.330)	0.000	1.402 (0.528, 2.277)	0.002
Lumen stenosis, %	0.282	0.080	−0.091 (−0.159, −0.023)	0.009	0.065 (−0.005, 0.136)	0.068
Minimal stent area (mm^2^)	0.292	0.085	0.729 (0.207, 1.252)	0.007	−0.473 (−1.411, 0.465)	0.318
Minimum stent expansion, %	0.365	0.134	−0.092 (−0.144, −0.041)	0.001	0.082 (−0.049, 0.213)	0.218
Mean stent expansion, %	0.376	0.141	−0.079 (−0.122, −0.037)	0.000	−0.014 (−0.102, 0.074)	0.753
Lumen volume (mm^3^)	0.192	0.037	−0.026 (−0.054, 0.003)	0.078		
Stent volume (mm^3^)	0.300	0.090	−0.034 (−0.057, −0.010)	0.005	−0.026 (−0.053, 0.001)	0.059
NIH volume (mm^3^)	0.339	0.115	−0.168 (−0.270, −0.066)	0.002	0.112 (−0.028, 0.252)	0.116
Malapposition volume (mm^3^)	0.685	0.469	0.715 (0.549, 0.881)	0.000	0.152 (−0.058, 0.362)	0.154
Difference between reference lumen area and mean stent area (mm^2^)	0.491	0.241	0.326 (0.199, 0.452)	0.491	1.418 (0.693, 2.143)	0.000

NIH—Neointimal hyperplasia.
